# Slowing after Observed Error Transfers across Tasks

**DOI:** 10.1371/journal.pone.0149836

**Published:** 2016-03-02

**Authors:** Lijun Wang, Weigang Pan, Jinfeng Tan, Congcong Liu, Antao Chen

**Affiliations:** Key Laboratory of Cognition and Personality of Ministry of Education, Faculty of Psychology, Southwest University, Chongqing 400715, China; ghent university, BELGIUM

## Abstract

After committing an error, participants tend to perform more slowly. This phenomenon is called post-error slowing (PES). Although previous studies have explored the PES effect in the context of observed errors, the issue as to whether the slowing effect generalizes across tasksets remains unclear. Further, the generation mechanisms of PES following observed errors must be examined. To address the above issues, we employed an observation-execution task in three experiments. During each trial, participants were required to mentally observe the outcomes of their partners in the observation task and then to perform their own key-press according to the mapping rules in the execution task. In Experiment 1, the same tasksets were utilized in the observation task and the execution task, and three error rate conditions (20%, 50% and 80%) were established in the observation task. The results revealed that the PES effect after observed errors was obtained in all three error rate conditions, replicating and extending previous studies. In Experiment 2, distinct stimuli and response rules were utilized in the observation task and the execution task. The result pattern was the same as that in Experiment 1, suggesting that the PES effect after observed errors was a generic adjustment process. In Experiment 3, the response deadline was shortened in the execution task to rule out the ceiling effect, and two error rate conditions (50% and 80%) were established in the observation task. The PES effect after observed errors was still obtained in the 50% and 80% error rate conditions. However, the accuracy in the post-observed error trials was comparable to that in the post-observed correct trials, suggesting that the slowing effect and improved accuracy did not rely on the same underlying mechanism. Current findings indicate that the occurrence of PES after observed errors is not dependent on the probability of observed errors, consistent with the assumption of cognitive control account. Moreover, the PES effect appears across tasksets with distinct stimuli and response rules in the context of observed errors, reflecting a generic process. Additionally, the slowing effect and improved accuracy in the post-observed error trial do not occur together, suggesting that they are independent behavioral adjustments in the context of observed errors.

## Introduction

In an ever-changing environment, human beings have to effectively monitor their behaviors to adjust and optimize future task performance according to experienced conflicts, errors or negative feedback. In addition to monitoring their own behaviors, people must be able to monitor and learn from others’ behaviors, especially others’ errors, to successfully adapt to social life. Post-error slowing (PES) is a typical behavioral adjustment phenomenon triggered by errors [1–5], which is normally quantified as the difference in reaction time (RT) between correct trials following errors and correct trials following correct trials [6].

The generation mechanism of the PES effect remains debatable [4,7–9]. The cognitive control account assumes that the PES effect is driven by error signals [10–12]. Following the commission of an error, participants may adopt a more cautious response strategy to avoid another error by decreasing activity in the response priming unit. Specifically, errors may enhance selective attention, leading to improved performance through slower response and improved accuracy after errors [3,13,14]. Moreover, several studies have reported improved accuracy after errors compared with post-correct trials [7,15–17]. Consistent with this account, Dutilh and colleagues used the drift diffusion model to isolate and identify the psychological processes responsible for the PES effect. They found that the PES effect was mainly associated with an increase in response caution [18].

An alternative account proposed by Notebaert and colleagues posits that the PES effect reflects the automatic orienting of attention toward infrequent errors [19]. When correct events are infrequent, post-correct slowing will occur. Thus, the slowing effect may result from the occupation of attentional resources and the time-consuming reorientation to the subsequent task. Along with slowing after errors, the detraction of attentional resources from task-relevant stimuli leads to a decrease of accuracy in post-error trials [8,19,20]. Similarly, Houtman and Notebaert demonstrated that participants had worse target detection in a rapid serial visual presentation immediately following an error in the flanker task [21].

Recently, studies using functional magnetic resonance imaging (fMRI) have consistently implicated the posterior medial frontal cortex (pMFC), including the anterior cingulate cortex (ACC), in the detection of one’s own errors and the regulation of the subsequent behaviors [22,23]. Interestingly, the pMFC is also activated when others’ errors are observed and detected [24,25]. Additionally, using electroencephalographic (EEG) techniques, error-related negativity (ERN), a typical error detection event-related potential component, is generated after the observation of someone else making an error [4,26–28]. These findings confirm that a common neural circuit is recruited for the monitoring of one’s own and others’ errors.

More importantly, several studies have confirmed that the PES effect can be triggered by others’ errors regardless of whether the other “individual” is a real partner [4,29,30]. Thus, an individual can internally understand the actions of the person with whom he is interacting, and then adjust his behaviors according to the other’s errors. This phenomenon is usually interpreted using the theory of the mirror-neuron mechanism [25,31,32], which emphasizes that the mirror neurons are activated when one acts and when one observes the same action performed by others [33–35]. However, it remains unclear whether behavioral adjustments are caused when one’s own action and those of others differ in the context of observed errors.

Concerning the context specificity of the post-error adjustment, prior investigations have resulted inconsistent findings. Cho et al. [36] provided initial support for generic control of error information and found slowing in post-error RT and improvement in post-error accuracy across different stimulus-response sets (the response rule remained consistent). In the task-switching paradigm, Forster and Cho [7] supported the above finding and showed that the PES effect generalized across task contexts and that greater PES effect coincided with greater improvement in post-error accuracy within the high error rate subsample. These findings suggest that post-error adjustment represents a strategic, control-mediated mechanism, supporting the cognitive control account. In contrast, research by Notebaert and Verguts [8] demonstrated that although the PES effect was observed when the stimulus set and/or the response rule differed between tasks, impaired accuracy following errors was observed. This finding provides evidence for the orienting account. Therefore, the investigation of post-observed error performance across task contexts may provide a deeper understanding of the generation mechanism of the PES effect.

In the present study, the PES effect was examined within and across the observation-execution task. Moreover, three error rate conditions (20/80, 50/50 and 80/20, correct/error) were established in the observation task. Previous studies have mainly focused on the influence of the low observation error rate because errors are usually rare in daily life, even in the laboratory environment. For example, Castellar and colleagues programmed the computer error rate of observed trials at 20% in each block [30]. However, the relatively low error rate contains both the error information and the event infrequency information. Whereas in the 50% error rate condition, the frequency information is controlled because the probability of observed error trials and observed correct trials is equal; in the 80% error rate condition, the error information is excluded in the infrequent correct trials. In this case, the role of error information and frequent information in the generation of the PES effect can be effectively investigated.

To address the above issues, we collected behavioral data from three independent samples. In Experiment 1, the observation task and the execution task were both the letter flanker stimuli [37]. For the observation-execution task, participants first observed the response of their partner in the observation task, and then performed their own response according to the mapping rule in the execution task during each trial. To determine whether the RT in the post-observed error trials increased, it was compared with the post-observed correct trials in the three observation error rates. Based on the cognitive control account [4,10], we can predict that the adjustment following observed errors may improve performance, including improved accuracy and slower subsequent responses in all three error rate conditions. However, according to the orienting account [8,19], we can predict a dissociated result pattern in the RT and accuracy in the trials following observed errors compared to trials following observed correct responses. The slowing effect after observed errors should be obtained only in the 20% error rate condition; the RTs on post-observed error trials and post-observed correct trials should be comparable in the 50% error rate condition; and an inverse result post-correct slowing should be obtained in the 80% error rate condition. However, decreased accuracy in the post-observed error trials should be obtained in all three error rate conditions.

In Experiment 2, the tasksets between the observation task and the execution task differed. The execution task was the same as in Experiment 1, but the letter flanker stimuli in the observation task were replaced by colored squares. If the PES effect after observed errors occurs across tasksets, we can conclude that the PES effect in the context of observed errors is a generalized adjustment; otherwise, it is a task-specific adjustment. Notably, if the PES effect was caused by the control-mediated mechanism, improved accuracy in the post-observed error trials should be obtained. In contrast, if the PES effect was the consequence of attentional orienting, impaired accuracy in the post-error trials should be obtained.

In Experiment 3, to rule out the potential influence of the ceiling effect in the accuracy of post-observed error trials, we increased the participants’ error rate by shortening the response deadline in the execution task. If the accuracy and slowing effect in the post-observed error trials do not occur together, we can conclude that the slowing effect after observed errors and the improvement of post-observed error accuracy are not due to the same underlying mechanism.

## Experiment 1

In this experiment, we programmed the same observation and execution taskset and employed 20%, 50% and 80% error rate conditions in the observation task.

### Materials and Methods

#### Ethics statement

Approval for the study was provided by the Human Research Ethics Committee of Southwest University of China, and all participants provided written informed consent. All data underlying the findings are fully available without restriction. All behavior data files are available from the figshare database.

#### Participants

Thirty-two healthy volunteers (23 females, mean age = 21.63 years, SD = 1.83, range: 18–25 years) were recruited to take part in this study. All participants were right-handed except for one female who was left-handed, and all participants had normal or corrected-to-normal vision. The data from four participants were excluded for insufficient trials for statistical analyses in the infrequent event conditions (i.e., the infrequent errors in the 20% error rate condition and the infrequent correct trials in the 80% error rate conditions) (infrequent trials < 15). Therefore, data from twenty-eight participants (20 females) were entered into the final analysis.

#### Apparatus and Stimuli

The participants sat approximately 60 cm from a 17-inch monitor of a Lenovo computer. All stimuli were presented using E-prime software (Psychology Software Tools, Inc., Pittsburgh, PA). The stimuli of the flanker task were four capital English letters (H, N, E, and R), which were presented in black on a grey background. In congruent trials, the central letter and the flankers specified the same response, such as NNNNN; in incongruent trials, the central letter and the flankers specified different responses, such as NNHNN. Congruent and incongruent trials were pseudo-randomly sequenced with equal frequency. Before the experiment, the participants were instructed to respond to the central letters as quickly and accurately as possible. The letter-response key mappings were 1, 2, 9, and 0 on horizontally arranged number keys on a standard keyboard. The four responses corresponding to each letter were counterbalanced across the participants. Specifically, for half of the participants, the four target letters (H, N, E, and R) were mapped on the 1 key (left middle finger), 2 key (left index finger), 9 key (right index finger), and 0 key (right middle finger), respectively. For the other half of the participants, the four target letters (H, N, E, and R) were mapped on the 0 key (right middle finger), 9 key (right index finger), 2 key (left index finger), and 1 key (left middle finger), respectively.

#### Experimental procedure and Task

To achieve the experimental context of observed errors, before the experiment, the participants were informed that their partners would perform the same task in another room outside the experimental chamber. Moreover, they could see each other’s responses on their own computer screens. In fact, to effectively manipulate the error probability in the observation task, all partners were fictitious, and their responses were simulated by the computer.

Each trial started with a black fixation cross for 300 ms, followed by a blank screen for 300–500 ms. The observation task was then presented. An array of five letters was randomly presented in the center of the screen for 700–1,000 ms, and participants mentally gave a response to the central letter but did not overtly make a key-press. Following this, the response set (four numbers) was presented on the screen for 1,000 ms with one number surrounded by a red outline, which indicated the fictitious partner’s response. Here, the participants needed to observe the correctness of the responses made by their partners and remember the outcomes, which they were required to report orally at the end of each trial. After completing the observation task, the participants needed to perform an execution task. A stimulus was presented for a maximum of 1,500 ms (and terminated after any response key was pressed within this interval), during which time the participants were required to press the corresponding key according to the prespecified mapping rules. After the stimulus disappeared, a blank screen was displayed for 1,000 ms. Next, an asterisk cue reminded the participants to orally report the results about their partners’ responses, which were recorded by the experimenter through a serial response box (SRBOX). The asterisk cue sign was terminated by a key press within 2,400 ms. Finally, a 800–1,000 ms blank screen was displayed.

Each participant completed one practice block (50 trials) followed by six experimental blocks (80 trials each, 480 trials in all), with a one-minute break between blocks. Only when accuracy in the practice block (both execution accuracy and oral report accuracy) exceeded 80% could the participants go on to perform the formal experiment. Notably, the error rate of the observation task was established at 50% in the practice block. In the experimental blocks, 20%, 50% and 80% error rate conditions were run in two different orders (50-20-80 and 50-80-20) across blocks. Participants first performed the experiment in the 50-20-80 order and then in the 50-80-20 order. After the completion of each block, accuracy in the execution task was presented for 1,000 ms to monitor the participants’ performance.

### Results

For the RT analysis, the incorrect trials (incorrect key-press in the execution task and/or incorrect judgment in the observation task) and outlier trials (RTs shorter than 300 ms and longer than 1,350 ms) were removed. Correct trials following errors in the execution task were also discarded to rule out the confusion that the slowing in trials was due to the participants’ own errors. In total, 14.55% of the trials were excluded.

**Fig 1:**
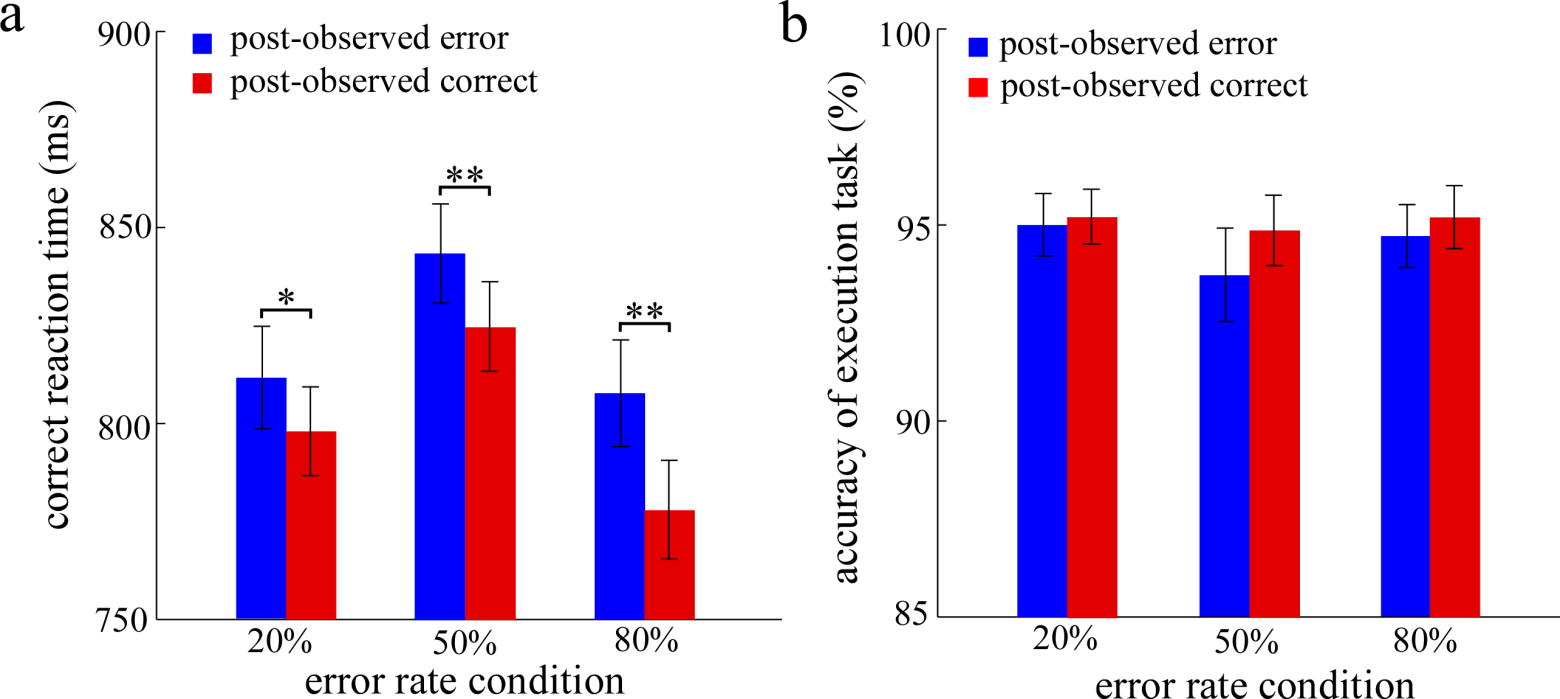
The results of Experiment 1. Panel a shows the mean reaction time for the trials of post-observed responses, and panel b shows the accuracy of post-observed responses. ms is milliseconds. Error bars denote standard error. Significant differences are indicated by asterisks (* *p* < 0.05, ** *p* < 0.01).

For the PES effect after observed errors, a repeated-measures analysis of variance (ANOVA) with response type (post-observed error and post-observed correct) and error rate condition (20%, 50% and 80%) as within-subjects factors was conducted (Fig 1A). The Greenhouse-Geisser correction was employed where appropriate. The results showed significant main effects of response type, *F*(1,27) = 49.34, *p* < 0.001, η_*p*_^2^ = 0.65, and error rate condition, *F*(2,54) = 25.75, *p* < 0.001, η_*p*_^2^ = 0.49. Moreover, the two-way interaction reached a significant level, *F*(2,54) = 4.11, *p* < 0.05, η_*p*_^2^ = 0.13. Post hoc tests revealed that RTs on post-observed error trials were significantly slower than those on post-observed correct trials in the three error rate conditions [20%: *F*(1,27) = 6.28, *p* < 0.05, η_*p*_^2^ = 0.19; 50%: *F*(1,27) = 25.26, *p* < 0.001, η_*p*_^2^ = 0.48; 80%: *F*(1,27) = 59.38, *p* < 0.001, η_*p*_^2^ = 0.69].

The result of an ANOVA with response type and error rate condition (50% and 20%) as factors revealed that the two-way interaction did not reach a significant level, *F*(1,27) < 1. However, the result of an ANOVA with response type and error rate condition (50% and 80%) as factors revealed that the two-way interaction was significant, *F*(1,27) = 4.45, *p* < 0.05, η_*p*_^2^ = 0.14.

To analyze the accuracy of post-observed responses, we employed an ANOVA with response type (post-observed error and post-observed correct trial) and error rate condition as within-subjects factors (Fig 1B). No significant result was found, *Fs* < 2.9. In addition, to ensure that the participants monitored their partners’ performance, the accuracy of the participants’ oral reports was analyzed. The results revealed the absence of significant main effects of response type and error rate condition, *Fs* < 1. However, the two-way interaction was significant, *F*(2,54) = 4.77, *p* < 0.05, η_*p*_^2^ = 0.15. Post hoc tests revealed that the accuracy of post-observed errors (96±0.5%) was significantly larger than the accuracy of post-observed correct trials (93±0.8%) in the 80% error rate condition, *F*(1,27) = 8.55, *p* < 0.05, η_*p*_^2^ = 0.24, but not in the 20% (observed error: 93.8±0.6% vs. observed correct: 94.2±0.6%) and 50% (observed error: 93.7±0.9% vs. observed correct: 94.5±1.1%) error rate conditions, *Fs* < 1.

### Discussion

RTs on post-observed errors were significantly slower than those on post-observed correct trials in all three error rate conditions. This result replicated and extended the findings of previous studies, confirming that the PES effect following observed errors occurred not only in the low observed error rate condition but also in the relatively high observed error rate conditions [4,30].

For accuracy, the results of the oral reports revealed that the participants’ performance in the observed trials was very accurate in the six experimental conditions, indicating that the participants did monitor their partners’ performance. However, it is likely that participants’ accuracy reaches a ceiling level in the execution task, which may lead the participants to fail to up-regulate their performance after observed errors. Thus, the accuracy was comparable in post-observed errors and post-observed correct trials.

Additionally, the interaction between response type and error rate condition (50% and 80%) was significant, but this did not occur when the error rate conditions were 50% and 20%. One possible reason for these results is that infrequent events enhance attention focusing on the target properties in the following trials. Specifically, the infrequent events were presented in observed correct trials in the 80% error rate condition, and enhanced attention focusing led to a faster response in the post-observed correct trials. Therefore, an increased PES effect following observed errors was obtained in the 80% error rate condition. However, the infrequent events were presented in observed error trials in the 20% error rate condition, and enhanced attention focusing might partly mask the slowing effect following observed errors. Accordingly, the PES effect following observed errors was comparable between the 20% and 50% error rate conditions. These results support the view that attention process induced by infrequent events is involved in the modulation of the PES effect following observed errors in a facilitative way [4].

## Experiment 2

To examine the task specificity of the PES effect in the context of observed errors, we programmed different tasksets in the observation and execution tasks. The execution task was the same as in Experiment 1, but the letter flanker stimuli in the observation task were replaced with colored squares. Therefore, the observation task and the execution task had different stimuli and stimulus-response mapping rules, but the response keys were the same across the two task types.

### Materials and Methods

#### Ethics statement

Approval for the study was provided by the Human Research Ethics Committee of Southwest University of China, and all participants provided written informed consent. All data underlying the findings are fully available without restriction. All behavior data files are available from the figshare database.

#### Participants

Thirty-three healthy volunteers (24 females, mean age = 22.39 years, SD = 2.05, range: 19–28 years) were recruited to take part in this study. All participants were right-handed and reported normal or corrected-to-normal full color vision. The data from five participants were excluded for insufficient trials for statistical analyses in the infrequent event conditions (i.e., the infrequent errors in the 20% error rate condition and the infrequent correct trials in the 80% error rate conditions) (infrequent trials < 15). Therefore, data from twenty-eight participants (20 females) were entered into the final analysis.

#### Apparatus, Stimuli, Procedure, and Task

The apparatus, stimuli, procedure and task were exactly the same as those used in Experiment 1, except that the stimuli of the observation task were replaced with the colored squares. The colored squares included red (255, 0, 0), green (0, 255, 0), yellow (255, 255, 0), and blue (0, 0, 255). Additionally, the stimulus-response mapping rules were as follows: red and the letter H were mapped on the 1 key (left middle finger), green and the letter N were mapped on the 2 key (left index finger), yellow and the letter E were mapped on the 9 key (right index finger), and blue and the letter R were mapped on the 0 key (right middle finger). The response rules were counterbalanced across the participants. For the other half of the participants, red and the letterr H were mapped on the 0 key (right middle finger), green and the letter N were mapped on the 9 key (right index finger), yellow and the letter E were mapped on the 2 key (left index finger), and blue and the letter R were mapped on the 1 key (left middle finger). Notably, the assigned responses in the observation task and the execution task in one trial were always different to avoid the response priming effect.

### Results

The data were analyzed in the same way as in Experiment 1. For the RT analysis, the incorrect trials (incorrect key-press in the execution task and/or incorrect judgment in the observation task) and outlier trials (RTs shorter than 300 ms and longer than 1,350 ms) were removed. Correct trials following errors in the execution task were also discarded to rule out the confusion that the slowing in trials was due to the participants’ own errors. In total, 16.54% of the trials were excluded.

**Fig 2:**
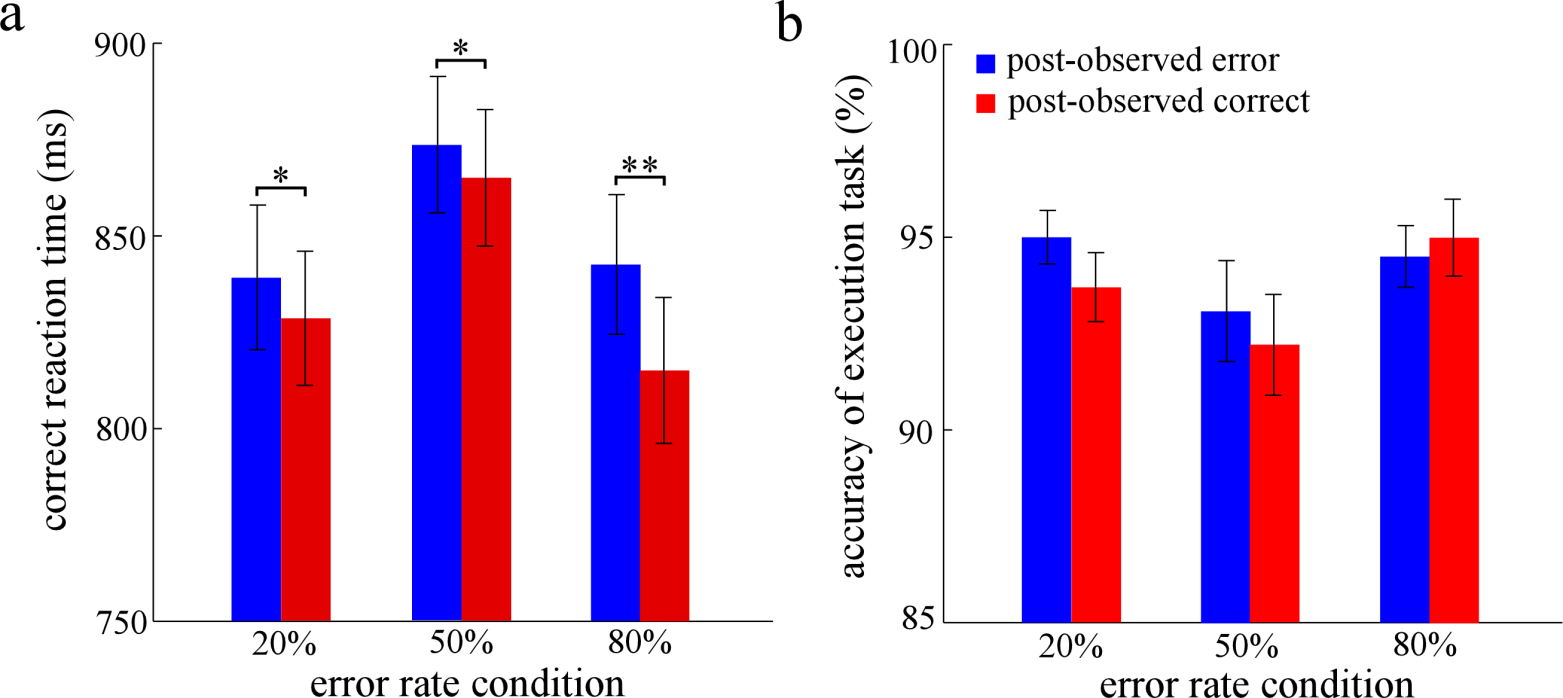
The results of Experiment 2. Panel a shows the mean reaction time for the trials of post-observed responses, and panel b shows the accuracy of post-observed responses. ms is milliseconds. Error bars denote standard error. Significant differences are indicated by asterisks (* *p* < 0.05, ** *p* < 0.01).

For the PES effect after observed errors (Fig 2A), the results showed significant main effects of response type, *F*(1, 27) = 31.28, *p* < 0.001, η_*p*_^2^ = 0.54, and error rate condition, *F*(2,54) = 53.48, *p* < 0.001, η_*p*_^2^ = 0.66. Importantly, a significant interaction between response type and error rate condition was found, *F*(2,54) = 4.59, *p* < 0.05, η_*p*_^2^ = 0.15. Post hoc tests revealed that RTs on post-observed error trials were significantly slower than those on post-observed correct trials in all three error rate conditions [20%: *F*(1, 27) = 4.37, *p* < 0.05, η_*p*_^2^ = 0.14; 50%: *F*(1, 27) = 4.47, *p* < 0.05, η_*p*_^2^ = 0.15; 80%: *F*(1, 27) = 25.70, *p* < 0.001, η_*p*_^2^ = 0.49].

The result of an ANOVA with response type and error rate condition (50% and 20%) as factors revealed that the two-way interaction did not reach a significant level, *F*(1,27) < 1. However, the result of an ANOVA with response type and error rate condition (50% and 80%) as factors revealed that the two-way interaction was significant, *F*(1,27) = 7.06, *p* < 0.05, η_*p*_^2^ = 0.21.

For the accuracy of post-observed responses (Fig 2B), ANOVA revealed that the main effect of error rate condition was significant, *F*(2,54) = 3.74, *p* < 0.05, η_*p*_^2^ = 0.12. However, the main effect of response type was not significant, *F* < 1. Importantly, the two-way interaction reached a marginally significant level, *F*(2,54) = 2.74, *p* = 0.07, η_*p*_^2^ = 0.09. Post hoc tests revealed that the accuracy between the post-observed error trials and the post-observed correct trials was not significantly different in three error rate conditions, *Fs* < 3. In addition, with regard to the accuracy of the participants’ oral reports, the results revealed that no significant result was found, *Fs* < 1.4.

### Discussion

Experiment 2 confirmed and expanded the findings of Experiment 1. The PES effect after observed errors was found in three error rate conditions, although the stimuli and stimuli-response rules were distinct between tasks. This finding suggests that the PES effect is not task-specific in the context of observed errors.

Moreover, the accuracy between post-observed error trials and post-observed correct trials was not significantly different in the three error rate conditions. To clarify whether the ceiling effect caused this effect, a control experiment was conducted.

## Experiment 3

To rule out the potential influence of ceiling effect on the post-observed error accuracy, we shortened the response deadline in the execution task to increase the participants’ error rate. Notably, to ensure that sufficient trials could be used for statistical analyses in the infrequent event condition and that the length of this experiment was the same as Experiments 1 and 2, we eliminated the 20% error rate condition and added a block to the remaining 50% and 80% error rate conditions.

### Materials and Methods

#### Ethics statement

Approval for the study was provided by the Human Research Ethics Committee of Southwest University of China, and all participants provided written informed consent. All data underlying the findings are fully available without restriction. All behavior data files are available from the figshare database.

#### Participants

Twenty-six healthy volunteers (21 females, mean age = 21.38 years, SD = 2.02, range: 19–26 years) were recruited to take part in this study. All participants were right-handed and reported normal or corrected-to-normal full color vision. The data from two participants were excluded for insufficient trials for statistical analyses in the infrequent event condition (i.e., the infrequent correct trials in the 80% error rate conditions) (infrequent trials < 15). Therefore, data from twenty-four participants (19 females) were entered into the final analysis.

#### Apparatus, Stimuli, Procedure, and Task

The apparatus, stimuli, procedure and task were exactly the same as those used in Experiment 2. In addition, the response deadline was shortened to 1,200 ms in the execution task, and two error rate conditions (50% and 80%) were established in the observation task. The performance of the two error rate conditions was counterbalanced across the participants. During the experiment, half of the participants were required first to perform the 50% error rate condition (3 blocks, 80 trials each), and then to perform the 80% error rate condition (3blocks, 80 trials each). The other half followed the opposite procedure.

### Results

For the RT analysis, the incorrect trials (wrong key-press in the execution task and/or wrong judgment in the observation task) were removed. Correct trials following errors in the execution task were also discarded to rule out the confusion that the slowing in trials was due to the participants’ own errors. In total, 22.05% of the trials were excluded.

**Fig 3:**
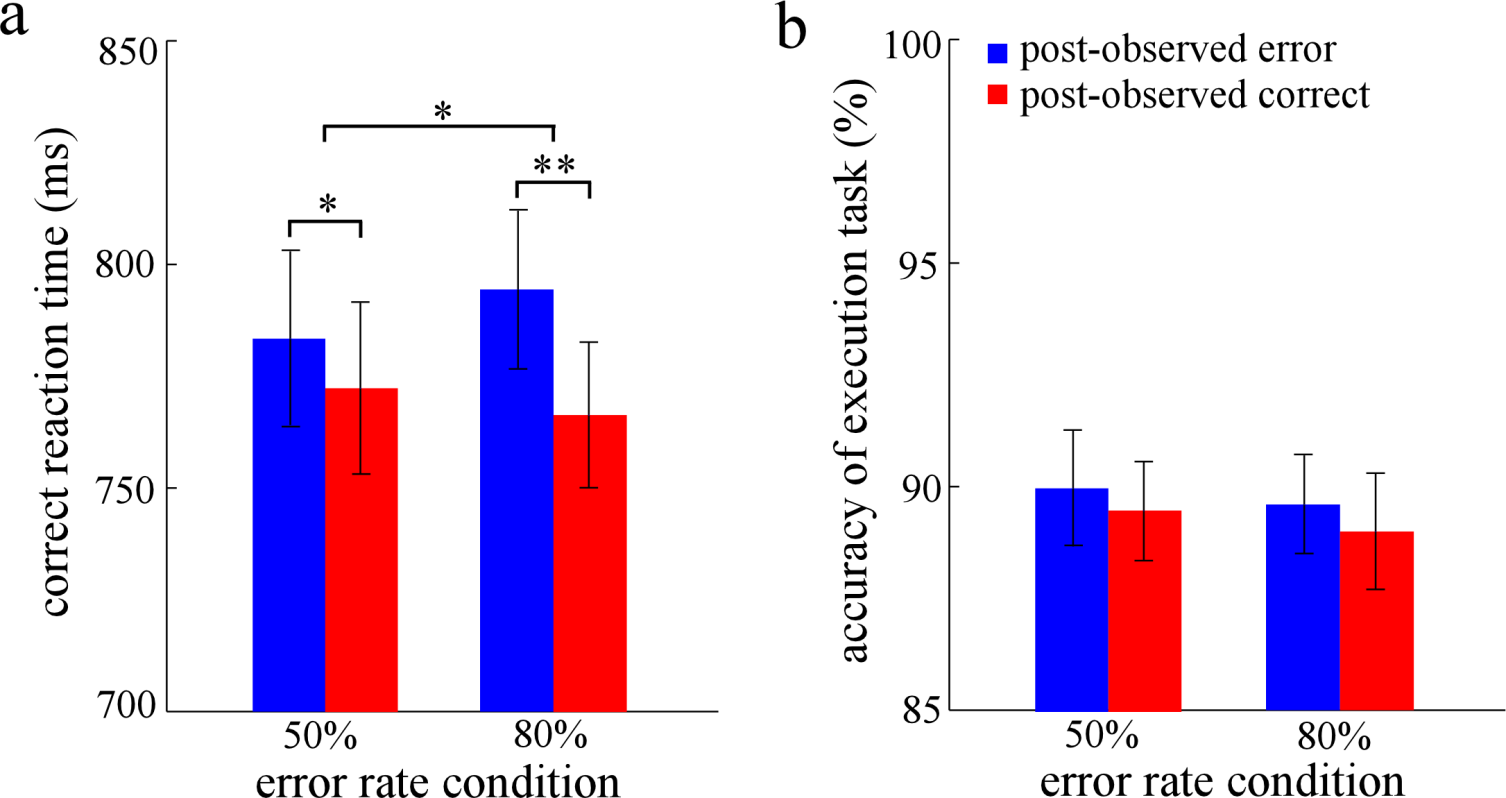
The results of Experiment 3. Panel a shows the mean reaction time for the trials of post-observed responses, and panel b shows the accuracy of post-observed responses. ms is milliseconds. Error bars denote standard error. Significant differences are indicated by asterisks (* *p* < 0.05, ** *p* < 0.01).

For the PES effect after observed errors (Fig 3A), ANOVA revealed that the main effect of response type was significant, *F*(1,23) = 29.35, *p* < 0.001, η_*p*_^2^ = 0.56. However, the main effect of error rate condition did not reach a significant level, *F* < 1. Importantly, the two-way interaction was significant, *F*(1,23) = 5.36, *p* < 0.05, η_*p*_^2^ = 0.19. Post hoc tests revealed that RTs on post-observed error trials were significantly slower than those on post-observed correct trials for the 50% [*F*(1,23) = 6.15, *p* < 0.05, η_*p*_^2^ = 0.21] and the 80% [*F*(1,23) = 22.72, *p* < 0.001, η_*p*_^2^ = 0.50] error rate conditions.

For the accuracy of post-observed responses (Fig 3B), the results revealed that no significant result was found, *Fs* < 1.3. For the oral report accuracy of observed responses, the main effect of response type was significant, *F*(1,23) = 37.84, *p* < 0.001, η_*p*_^2^ = 0.62, with greater oral report accuracy for the observed errors. However, neither the main effect of error rate condition nor the two-way interaction was significant, *Fs* < 1.

### Discussion

As in the Experiment 2, RTs in post-observed error trials were significantly slower than those in post-observed correct trials for both the 50% and 80% error rate conditions. However, although the participant’ error rate increased when the response deadline was shortened in the execution task, the accuracy between post-observed error trials and post-observed correct trials did not differ for the 50% and the 80% error rate conditions. This result was consistent with prior studies [11,38,39]. One possible reason is that current study design may lack sensitivity to detect the increased accuracy. Additionally, when the conflict event is detected, participants can adjust their subsequent behaviors in a goal-directed manner, such as enhancement in the goal-relevant dimension or inhibition in the goal-irrelevant dimension. However, the error events cannot provide the specific method of adjustment, indicating that the improved accuracy cannot be reliably obtained. Thus, the current findings may suggest that the improved accuracy and slowing effect in the post-observed error trials are independent adjustments [3,13].

## General Discussion

Using the observation-execution design, we investigated whether the PES effect was a generalized adjustment across tasks in the context of observed errors. Further, to best understand the underlying mechanism of the PES effect, we employed three error rate conditions (20%, 50% and 80%) in the observation task. In Experiment 1, the observation task and the execution task were both letter flanker stimuli. The results revealed that a significant PES effect after observed errors occurred in three conditions. In Experiment 2, the observation task was colored squares and the execution task was letter flanker stimuli. The result pattern was the same as Experiment 1, although the stimuli and stimuli-response rules were distinct across task contexts. In Experiment 3, an increased error rate was observed when the response deadline was shortened in the execution task. However, there was no difference between post-observed error and post-observed correct accuracy. These findings suggest that 1) the PES effect generalizes across tasks in the context of observed errors; 2) the improved accuracy and slowing effect in the post-observed trials are independent adjustments following observed errors; and 3) the PES effect reflects a strategic, control-mediated mechanism.

RTs on correct trials following observed errors tended to be slower than those following observed correct responses in all three error rate conditions. This result replicates and extends the findings of prior studies [29,30], suggesting that the occurance of a slowing effect after observed errors does not depend on the probability of observed errors. The cognitive control account states that error information in a preceding trial causes participants to adopt a more cautious strategy [7,10,18], leading to prolonged RT on the subsequent trial. Thus, the slowing effect after errors should be obtained in all post-error trials. The current results were in accordance with the above assumption. Particularly, since the frequency information was controlled in the 50% error rate condition, the PES effect after observed errors was obtained in this condition supporting that cognitive control induced by error information plays an important role in the generation of the PES effect following observed errors.

Considering that observed errors involve comparison between the representations of the partner’s goal response and the representations of the partner’s actual response [4,29], the mismatch theory may provide an alternative explanation [40]. More specifically, one’s own estimation of the partner’s goal response is not confirmed by the partner’s response, leading to a mismatch. This mismatch signal will initiate the remedial action system, which is responsible for inhibiting or correcting the error [40]. Therefore, less time is available to prepare for the forthcoming target response after a mismatch, leading to a slower response to mismatch evaluation than to match evaluation. The mismatch signal is not up to the error frequency.

Interestingly, when stimuli and stimuli-response rules were respectively established in the observation task and the execution task, the robust PES effect was found across tasksets. This result may suggest that the occurrence of the PES effect translates into generic post-error adjustment across distinct task demands and response sets in the context of observed errors. However, Picton et al. [41] instructed two participants to perform a joint choice-RT task based on the same stimulus but different response rules. The results revealed that participants monitored others’ errors but could not adjust their subsequent performance according to the partners’ errors, in contrast to the current findings. It is likely that when two participants perform tasks simultaneously, they focus on their own results first. In this case, monitoring the other’s performance is insufficient to trigger one’s own behavioral adjustment. However, in the observation-execution task, participants were required to first observe the outcomes of their partners, and then to perform their own task. Thus, the participants could utilize the error information from their partner’s outcomes to adjust their own behavior, even in the context of task switching.

Evidence from Notebeart and Verguts [8] supports the view that the PES effect is a general process across tasksets. However, these authors find that accuracy tends to decrease after errors in three of four task conditions and find a marginally significant correlation between individual’s error rate and the PES effect, supporting the orienting account. However, research by Forster and Cho [7] reveals that greater PES effect coincids with greater improvement in post-error accuracy within the high error rate subsample, providing evidence for the cognitive control account. In contrast, we found that accuracy between the post-error trials and the post-correct trials was comparable in three error rate conditions in the context of observed errors. The possible reasons are as follows. First, the error types are different. Observed errors in the present study may reduce the motivational significance of errors, leading to a decrease in the activation of the response units without improved accuracy in the post-observed error trials. Second, the error events cannot provide the specific method of adjustment to the subsequent task, such as enhancing the goal-relevant process or inhibiting the goal-irrelevant process. In this case, the adjustment strategy may slow down the subsequent task but not increase the accuracy. Third, the improvement in post-error accuracy and the PES effect do not always occur together and are not necessarily correlated [3,11,38,39]. For example, Dudschig and Jentzsch [42] investigated the influence of response-stimulus intervals on RT and accuracy in post-error trials and found increased PES effect and reduced post-error accuracy with decreasing response-stimulus intervals, suggesting that post-error adjustments followed different time courses [43]. Therefore, the slowing effect following observed errors may be independent from improved accuracy in the post-observed error trials.

In addition, the magnitude of PES effect was not significant between the 20% and the 50% error rate conditions, whereas the magnitude of PES effect was significantly larger for the 80% error rate condition than for the 50% error rate condition. Infrequent events may enhance the attentional focus on goal-related information, leading to faster response in trials following infrequent events. In this case, faster post-observed correct response was obtained in the 80% error rate condition, leading to an increased PES effect, whereas the slowing effect after observed errors was partly masked in the 20% error rate condition, leading to an equivalent PES effect between the 20% and the 50% error rate conditions. The current results suggest that frequency information is also involved in the generation of PES effect in a facilitative way. Therefore, these findings support the view that attention process induced by frequency information contributes to the modulation of the magnitude of PES effect following observed errors [4].

However, the current results were inconsistent with the orienting account. There are multiple reasons for these inconsistent findings among the studies. First, the task context of observed errors influences the PES effect differently. Several studies have affirmed that social context can modulate the error processing [5,29]. Second, the task configurations between the two lines of study are different. The performance in the color discrimination task may rely heavily on visual input. In other words, bottom-up attention process influences this task performance. However, the flanker task requires participants to focus on a task-relevant dimension and to inhibit the task-irrelevant dimension. Thus, the flanker task may rely on top-down attention modulation. Third, this finding may be linked to different error types. Errors in the study of Notebaert and his colleagues [19] were primarily caused by encoding failures and fast guessing during the stimuli, due to stimulus ambiguity [7]. Observed errors in the present study were caused by the inconsistency between the representations of the correct response and the representations of the partner’s actual response [4,29].

This study may have some limitations. First, one might argue that the high observation error rate was meaningless. The 80% error rate in the observation task implied that the performance by the partner was worse than random. In this case, one might choose to ignore the partner’s outcomes. However, we adopted a forced-choice test (in a way of oral report) to ensure that participants accurately monitored others’ outcomes in this study. Additionally, incorrect estimations of the partner’s outcomes were removed before the statistical analysis. Thus, we believe that our design could successfully manipulate the participants’ degree of task involvement during the observation task. Second, to help record the participants’ oral reports, an experimenter needed to stay with the participants in a room. Whether the experimenter and the participant are the same sex or opposite sexes may be an additional variable. Future studies should aim to investigate the underlying mechanisms of the PES effect following observed errors with better ecological validity. Third, for completing the oral report, participants needed to keep the oral response in mind. Thus, the slower responses in the execution task might be caused by interference from the oral report. To examine whether the interference was a potential source of the slower responses, we conducted another control experiment. In this experiment, participants were instructed to observe the outcomes of their partners but not to provide an oral report. When the experiment was completed, the participants needed to answer whether they could estimate the range of the probability of errors made by their partner (participants did not know this before the experiment). This experiment included only the 80% error rate condition. The results revealed that the slower effect following the observed errors was still obtained. Thus, the slower effect could not be attribute to the interference of oral report (please see the supplemental materials for more detail).

## Conclusions

The current results confirm that the occurrence of the PES effect after observed errors does not depend on the probability of observed errors. Moreover, increased PES in the 80% error rate condition and impaired PES in the 20% error rate condition suggest that frequency information is also involved (in a facilitating manner) in the generation of the PES effect after observed errors. Additionally, the PES effect represents a generic adjustment process across distinct task demands and stimuli-response rules in the context of observed errors. The current findings favor the cognitive control account that posits the PES effect is a strategic adaptive modulation driven by error information. Notably, the post-observed error accuracy was comparable to the post-observed correct accuracy in three error rate conditions, even when the error rate was increased in the execution task, suggesting that the slowing effect and improved accuracy in the post-observed error trials may not be due to the same underlying mechanism.

## Supporting Information

S1 Text The control experiment investigating whether the interference of oral report was a potential source of the slower responses. (DOC)
