# Supplementary material for: Slowing after Observed Error Transfers across Tasks
Source: PLoS One. 2016 Mar 2;11(3):e0149836. doi: 10.1371/journal.pone.0149836 (PMC4775031; doi:10.1371/journal.pone.0149836)
Supplement: S1 Text — (DOC) [file pone.0149836.s001.doc]

The instruction given to the participants to orally report the outcomes of observed responses made by their partners at the end of each trial might interfere with their performance in the execution task. To examine whether the slower effect was caused by the interference of the oral report, we excluded this instruction and required participants to observe the outcomes of their partners. Additionally, to ensure that participants did monitor their partners’ performance, a question was asked at the end of the experiment (participants did not know this before the experiment). The question was that “Can you estimate the range of the probability of errors made by your partner”. This experiment only included the 80% error rate condition. If the participants answered the range was 70%-90%, we believed that they accurately monitored their partners’ performance. Because previous studies have reported that the PES effect still be obtained when there is no explicit instruction of monitoring the partner’s performance (e.g. to count the observation errors; [1,2]). We predicted that the PES effect following observed errors should be obtained without an oral report.

**Materials and Methods**

**Ethics statement**

Approval for the study was provided by the Human Research Ethics Committee of Southwest University of China, and all participants provided written informed consent. All data underlying the findings are fully available without restriction. All behavior data files are available from the figshare database.

**Participants**

Twenty-eight healthy volunteers (20 females, mean age = 22.85 years, SD = 1.63, range: 18-26 years) were recruited to take part in this study. All participants were right-handed and had normal or corrected-to-normal vision. The data from four participants had to be excluded for the following reasons: poor behavioral performance (1 participant) and incorrect estimation of range (3 participants). Therefore, data from twenty-four participants (17 females) were entered into the final analysis.

**Apparatus, Stimuli, and Task**

These were exactly the same as those used in Experiment 1 except that only 80% error rate was employed in the observation task. In addition, as illustrated above, the explicit instruction given participants to monitor their partners’ performance was removed. Moreover, after completing the experiment, the participants were required to estimate the range of errors committed by the partners to ensure participants participated in the observation task.

**Results**

For the RT analysis, the incorrect trials (incorrect key-press in the execution task and/or incorrect judgment in the observation task) and outlier trials (more than three standard deviations from the mean RT) were excluded. Correct trials following errors in the execution task were also discarded to rule out the confusion that the slowing in trials was due to the participants’ own errors. In total, 20.80% of trials were removed. The paired-samples *t* test was used to analyze the PES effect following observed errors. The result revealed that RTs on post-observed error trials were significantly slower than those on post-observed correct trials, *t*_23_ = 2.05, *p* < 0.05. The results of analysis of the accuracy of post-observed responses revealed that there was no difference between post-observed errors and post-observed correct trials, *t*_23_ = 1.15, *p* > 0.1.

**Discussion**

When participants were required just to observe but not to do anything else in the observation task, significant PES effect following observed errors still occurred in the 80% error rate condition. This result suggested that the slowing in the observed context was not the result of interference from the oral report, paralleling with the findings of previous studies [1,2].

**References**

1. Núñez Castellar E, Notebaert W, Van den Bossche L, Fias W. How monitoring other’s actions influences one’s own performance: post-error adjustments are influenced by the nature of the social interaction. Exp Psychol. 2011;58: 499-508.

2. Schuch S, Tipper SP. On observing another person’s actions: Influences of observed inhibition and errors. Percept Psychophys. 2007;69: 828-837.
